# Corrosive oesophageal injuries: a preventable menace

**DOI:** 10.11604/pamj.2013.15.11.2495

**Published:** 2013-05-06

**Authors:** Taiwo Olugbemiga Adedeji, James Enajero Tobih, Adedayo Olugbenga Olaosun, Olusola Ayodele Sogebi

**Affiliations:** 1 Department of Ear Nose and Throat, LAUTECH Teaching Hospital, Osogbo, Osun state, Nigeria; 2 Department of Ear Nose and Throat Olabisi Onabanjo University Teaching Hospital, Ago Iwoye, Ogun State, Nigeria

**Keywords:** Corrosive injuries, caustic ingestion, accidental ingestion, self harm, mechanisms, prevention, psychiatric disorders, esophageal stricture, Nigeria

## Abstract

**Introduction:**

Potentially catastrophic presentations and lifelong complications resulting from corrosive ingestions in humans is one of the most challenging situations encountered in clinical medical practice. This study reviewed pattern, mechanisms and associated socio-medical challenges with ingestion of corrosive agents as seen in a tertiary health institution in South-western Nigeria.

**Methods:**

A retrospective review of all patients that were managed for corrosive ingestion at Ladoke Akintola University of Technology Teaching Hospital, Osogbo, Osun State, Nigeria, over a seven year period.

**Results:**

A total of 28 patients M:F: 1.6:1. There were 7 children and 21 adults. Majority (78.6%) of the patients ingested alkaline substances. Accidental ingestion occurred in 28.6% while 71.4% resulted from deliberate self harm especially among adults (66.7%). Almost two thirds (64.3%) of the patients presented after 48hrs of ingestion. Patients who presented early were managed conservatively. Most patients (64.3%) who presented late had nutritional and fluid rehabilitation. Two patients died from oesophageal perforation and resulting septicaemia. Psychiatric evaluation revealed that seven adults (25%) had psychotic illness while (42.9%) of the patients developed oesophageal strictures. Short segment strictures were managed with oesophageal dilatation with good outcome while long and multiple segment strictures were referred to cardiothoracic surgeons for management.

**Conclusion:**

Corrosive oesophageal injuries remain a prevalent and preventable condition in the developing countries. Preventive strategies should include regulation and packaging of corrosive substances, organization of psychiatric services, and education of the population on corrosive ingestion.

## Introduction

Potentially catastrophic presentations and lifelong complications resulting from caustic ingestions in humans is one of the most challenging situations encountered in clinical medical practice [1–3]. Corrosive ingestion constitutes 0.3% of paediatric admission in the Gambia [4] and 0.5% in Nigeria [5], and was responsible for 0.8% of total childhood mortality in Gambia [6].

Caustic material ingestion is most frequently accidental in children particularly those from families with low income [7–9]. In adults, corrosives are usually ingested either for suicidal or for medicinal purposes [2, 7]. In this population, the injuries are often more serious because they are intentional [3]. The ingested chemicals which could either be an acid, or an alkali/ base, have high corrosive potentials.

In households, caustic soda is used for making soap by traditional/local methods in many poor countries [3]. Caustic soda inside bottles can be confused with water or alcoholic beverages and can be ingested accidentally [3]. The ingestion leads to destruction of tissue which can result in complications such as respiratory distress, oesophageal and gastric perforations, septicaemia and death [2]. The degree and extent of corrosive lesion and its complications depend on several factors such as concentration of caustic substance, quantity swallowed, fullness of the stomach and duration of contact with tissue or organs [6, 10] and the quality of care given at the initial management of the patient at presentation. In many cases, stricture formation is inevitable and long term risk of developing cancer of oesophagus is higher among those affected than in the normal population [2, 10]. In the western world, the common causes of benign oesophageal stricture are hiatal hernia and reflux esophagitis but in Nigeria, the most common cause of benign oesophageal stricture is ingestion of corrosive [7].

Management of corrosive ingestion and its sequel constitute a medical challenge to the Otolaryngologist. Contini et al. [6] reported that majority of oesophageal caustic strictures in developing countries usually presented late when dilation procedures are likely to be more difficult and carry significant high recurrence rate [6]. This late presentation may be related to ignorance on the nature of the disease and its management and also to poverty [10, 11]. Patients initially seek home-based therapy by traditional healers7 and only present at the hospital after complications had set in. Furthermore, some patients also default at the follow up clinic [11–13].

Legislation to limit the concentration of hazardous cleaners and to ensure that containers are child-proof has been advocated for a long time [1]. In the western world, the incidence of corrosive esophageal injuries has declined due to legislative effort and stricter packaging standards [3, 13]. Unfortunately this may not be the situation in the developing countries [6]. The dearth of literature in our environment on this subject stimulated our interest to examine pattern, mechanisms and associated socio-medical challenges associated with ingestion of corrosive agents as seen in a tertiary health institution in Osogbo, South-western Nigeria

## Methods

The study was a retrospective review of all patients that were admitted and managed for corrosive ingestion at Ladoke Akintola University of Technology Teaching Hospital, Osogbo, Osun State, Nigeria, over a seven year period between 2005 and 2011. The case notes of the patients were retrieved from the medical records department of the hospital. Data retrieved from the case records included patients' age, sex, type of corrosive ingested, duration between corrosive ingestion and presentation at the hospital, reason(s) for ingesting corrosive agent, treatment modality and complications. Excluded were patients whose case records could not be located and those that had incomplete information. The information was entered into a spread sheet and analysed using SPSS version 14. The data was presented in simple descriptive forms as proportions using tables and graphic chart.

## Results

A total of 28 patients with corrosive ingestion were admitted and managed during the period of study. There were 17 male and 11 females (M: F: 1.6:1). The patients were aged between 2 and 75 years, with a mean age of 32.1 years. There were 7 children and 21 adults.

Accidental ingestion occurred in 8 (28.6%) patients while in 20 (71.4%) patients ingested for deliberate self harm. All the affected children were due to accidental ingestion and majority (5/7 (71.4%)) of them occurred while playing in their neighbour's houses where caustic soda was used traditionally for soap making. Most (66.7 %) of the affected adults were due to deliberate suicidal attempts. Ten (35.7%) patients presented within the first 48hours (regarded as early presentation) while other patients presented after the first 48 hours (late presentation). Details of the clinical characteristics are shown in Table 1.

**Table 1:** Clinical characteristics of the patients

Variable		Frequency	Percentage
Age range (in years)	1-15	7	25.0
	16-30	8	28.6
	31-45	6	21.4
	46-60	4	14.3
	61 and above	3	10.7
Sex	Male	17	60.7
	Female	11	39.3
Mechanism of injury:	Accidental	8	28.6
	Deliberate self-harm	20	71.4
Duration of symptoms (hrs)	0-48	10	35.7
	>48	18	64.3

**Figure 1:**
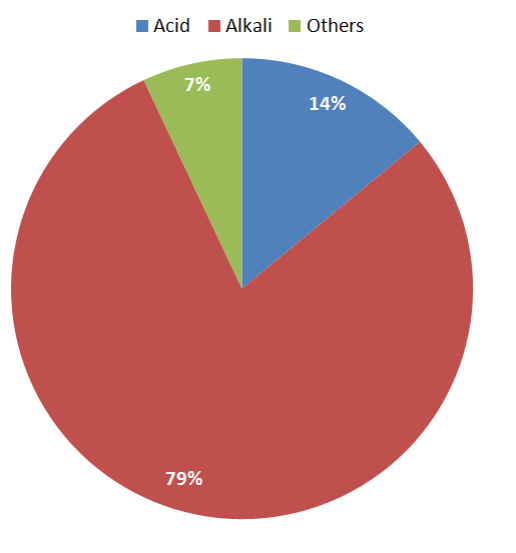
Pie chart showing the nature of corrosive agents

Figure 1 depicts the percentage distribution of the nature of ingested substances; four patients (14.3%) ingested acid, 22 (78.6%) ingested alkali while 2 (7.14%) patients ingested other corrosive agents. Most patients (64.3%) presented late and two (7.1%) mortalities were recorded. Psychiatric evaluation revealed that seven (33.3% of adults) had depressive/psychotic illness. Twelve patients (42.9%) developed oesophageal stricture, (58.3%) of which were long or multiple segments) seen in Table 2. Three of these patients with stricture initially discharged themselves against medical advice and only re- presented when stricture had developed in them. Short segment strictures were managed with oesophageal dilatation while long or multiple segment strictures were referred to cardiothoracic surgical unit for possible oesophageal replacement

**Table 2:** Associated complications of corrosive oesophageal injuries

Complications	Number of patients	Percent
Nil	11	39.3
Malnutrition	13	46.4
Oesophageal stricture	12	42.9
Laryngitis	2	7.2
Died	2	7.1
**Total**	**28**	**100.0**

## Discussion

Accidental ingestion of corrosive substances is declining in developed countries [13] but not in developing countries, where it is relatively common especially among the illiterates with poor socioeconomic status [6, 17–20]. In this study ingestion of corrosives is the most common cause of oesophageal stricture. This is in agreement with previous reports over a decade ago in neighbouring Ibadan on the subject [7]. This presupposes that unregulated usage of corrosive substances is still rampant in our environment and its sequel has not changed. A study from South Africa reported that corrosive agent was readily available and within reach of the affected patients [21]. Johnson et al [13] however reported that legislative effort had significantly reduced the incidence of paediatric corrosive ingestion in the USA. Majority (78.6%) of the patients in our study ingested alkali, which is the chemical used in making soap by empirical means in most poor homes [6]. Many studies [1, 6, 13, 20] reported that ingestion of alkali is more prevalent than that of acid in corrosive oesophageal injuries. Thomas et al. [9] however reported a contrary finding. Alkali is usually kept in containers without sealed covers by people that manufacture local soaps; accidental ingestion from mistaken identity was the mechanism of ingestion in all the children in this study. Majority of the affected children confused these chemicals with water while playing in neighbourhoods or even in their own houses.

Most of the adults deliberately took corrosives with the intent of committing suicide or deliberate self harm as the agents were readily available as reported in previous literature [12, 14, 16]. None or ineffective regulation of potentially harmful chemical agents makes the environment unsafe and constitutes substantial risk to both children and adults [6]. Corrosive ingestion is a significant burden especially among illiterates with poor socioeconomic status [18–20]. This group of people should be targets for education about creating a safe environment by regulation of chemicals. Education will increase awareness of dangers associated with corrosive ingestion so as to guard against indiscriminate placement of corrosive agents, child resistant / child proof packaging will also prevent easy access to corrosive agents by children. This information will help in prevention of these common accidents in children as well as adults.

Another important finding from this study was that some of the affected adults were on psychiatric evaluation, discovered with depressive or psychotic illness that predisposed them to suicidal/ para-suicidal tendencies. Wilson et al [14] in their study reported that 33% of patients that ingested corrosives had psychiatric disorders that warranted medication and that 11% had acted on psychiatric belief. Other studies also showed that 29 - 90 % 0f patients who ingested corrosives had psychotic disorders [21, 22]. In this group of individuals, corrosive ingestion would have been possibly prevented by good family support or periodic psychiatric evaluation.

Poverty was also noted to be a contributory factor in corrosive ingestion. The two mortalities recorded in this study were those of secondary school students who became frustrated and subsequently ingested corrosives because their parents could not afford their school examination fees. Poverty eradication program of the government should be stepped up to reduce frustrations people face with resultant depressive/ psychological trauma. This suicidal tendencies and ready availability of corrosive substances created an enabling environment for its ingestion [19–21]. Other implications of corrosive ingestion includes economic burden of medical treatment, tendencies to psychiatric disorders on the patients and general reduction in their qualities of life had been reported [10, 12, 13]. It is obvious that although caustic materials may be useful, it also constitutes a potential public health risk [1] and hence its use needs to be regulated to reduce or possibly prevent its ingestion.

The fact that majority of the adults ingested corrosives for deliberate self harm especially suicide is disturbing; some of the patients have been confirmed with psychiatric illnesses, possibly aggravated by low socio-economic status. There is need for a good social support system to assist citizens with low economic empowerment which will assist in relieving some of the stresses that predispose such adults to suicidal tendencies. Psychiatric evaluation of adults should be taken serious and introduced down to the primary health care level while stigmatization associated with psychiatric illnesses in our environment should be discouraged.

Almost half (42.9%) of the affected patients developed oesophageal stricture which may be attributable to various factors. Mamede et al. [17] reported 65.3% oesophageal stricture following corrosive ingestion. The factors responsible for stricture development include mechanism of injury, especially deliberate self harm, nature of ingested chemical time of presentation [17, 20] as well as the methods of management at the initial presentation. Majority 79.0% of our patients ingested alkalis, which cause liquefactive necrosis of the oesophagus and associated with more extensive mucosa disease and oesophageal perforations and complications, in comparison with acids which cause coagualtive necrosis. Patients that presented early were managed conservatively i.e. nasogastric tube passage for stenting and for feeding; intravenous fluid, antibiotics; analgesic; antacids in form of H2- receptor blocker and proton pump inhibitors. Steroid was not routinely used. This line of management has been found to prevent complications like malnutrition, electrolyte imbalance and reduction in the tendency to develop stricture formation [8].

Corrosive oesophageal injuries in developing countries have been observed to present late with established strictures which is associated with poorer outcome on dilatation, recurrence and generally poor prognosis [6]. Furthermore, late presentation is reported to be a strong predictor for future oesophageal replacement [6].

Patients that deliberately or intentionally ingested corrosives were prone to develop severe injuries. Qureshi et al. [23] reported that corrosive injuries are frequently more serious in adults who intentionally ingested chemicals and usually in large volumes which leads to lifelong debilitating conditions. Most patients that developed strictures ingested the corrosives with the intent of deliberate self harm. Ingestion of alkali causes more injury to the oesophagus when compared to acid [1]. Alkali tends to affect the body of the oesophagus, especially at areas of natural constrictions [6, 19] like the cricopharyngeal junction, etc. and cause extensive damage. Furthermore the attitude of some of the patients contributed to the poor outcome of disease. For instance, some patients discharged themselves against medical advice only to re-present when stricture had developed and will be difficult to manage [6] as it happened in three patients in this study. This may be connected with ignorance on the part of the patients and consequently affected the outcome. The need for education especially on the nature, management and possible outcome of the disease both for the patients and their relatives cannot be overemphasized.

## Conclusion

In conclusion, this study found that corrosives ingestion is still common and many resulted from deliberate self harm in adults with psychiatric tendencies. Most patients presented late when complications had developed. Oesophageal corrosives ingestion remains a preventable disease and the methods of prevention were discussed.
